# Electronic Mental Health as an Option for Egyptian Psychiatry: Cross-Sectional Study

**DOI:** 10.2196/19591

**Published:** 2020-08-13

**Authors:** Mostafa Mamdouh Kamel, Jean Nicolas Westenberg, Fiona Choi, Katarina Tabi, Adel Badawy, Hisham Ramy, Hossam Elsawi, Michael Krausz

**Affiliations:** 1 Department of Psychiatry University of British Columbia Vancouver, BC Canada; 2 Department of Psychiatry Tanta University Tanta Egypt; 3 Healthy Minds Centre BC Children's Hospital Vancouver, BC Canada; 4 Institute of Psychiatry Ain Shams University Cairo Egypt

**Keywords:** psychiatry, e-mental health, Arab countries, mental health care, psychiatrists, health care providers

## Abstract

**Background:**

Egypt is a country of nearly 100 million citizens, and there are less than 1000 registered psychiatrists. The mental health care system is under resourced and nearly inaccessible for the majority of the population. In addition, youth under the age of 25 years represent 50% of Egyptian citizens; however, there are no specific services addressing their unique needs. How can the needs of the largest population in the Middle East be effectively addressed? Is a web-based framework an option for Egyptian psychiatrists to serve the population?

**Objective:**

The aims of this study were to better understand the opinions of psychiatrists on the current state of mental health care services in Egypt and their current knowledge on electronic mental health (EMH); assess the attitudes of Egyptian psychiatrists toward web-based interventions and telemedicine for mental health; and identify perceived advantages and barriers of EMH development in Egypt.

**Methods:**

A cross-sectional survey was conducted online among 640 Egyptian psychiatrists. It included a total of 36 items within a set of 16 questions asking about EMH literacy, integrating EMH into the mental health care system, and the perceived priorities and barriers of EMH. The sampling was supported by Tanta University, a large academic institution close to Cairo. Statistical analysis was performed using SPSS 25 (IBM Corp). Descriptive statistics, the chi-square test, the independent sample *t* test, and analysis of variance were applied.

**Results:**

A total of 188 participants responded (response rate of 29.4%), of which 54.2% (102/188) were female and 54.3% (102/188) were between 30 and 45 years old. Less than half of the participants thought that the current health care system was efficient for adults (69/155, 44.4%), and even less thought it was efficient for youth (44/155, 28.3%). Almost all participants agreed that EMH would be beneficial for patient care (147/155, 94.8%) and that integrating EMH into the current health care system would be a good idea (118/155, 76.2%). The highest rated utility of web-based solutions was documentation, followed by psychoeducation and communication with professionals. The main advantages were to improve access to care in rural areas of the country and its convenience.

**Conclusions:**

There is scarcity of mental health resources in Egypt. Egyptian psychiatrists are interested in EMH and believe web-based platforms can become part of the solution for the Egyptian mental health care system.

## Introduction

Arab countries generally have a higher burden of mental health disorders, as measured by the disability-adjusted life years, relative to the rest of the world [1,2]. In fact, of all the countries within the Eastern Mediterranean Region, Egypt is the only country to have a burden of mental illness equivalent to the global level [3]. The General Health Questionnaire administered to over 25,000 individuals in Egypt estimated a prevalence of 24.9% for psychiatric comorbidity, and these mainly included mood (depressive), anxiety, and substance use disorders [4]. Importantly, people with mental illness in Egypt and other Arab countries tend to somaticize their psychological symptoms and often seek traditional healers for most types of mental and physical health problems owing to societal acceptance, affordability, and accessibility [5,6]. Stigmatizing attitudes toward people with mental illness contributes to a reluctance to seek psychiatric care, and similarly, expensive and inaccessible psychiatric care deters individuals from the mental health system, which is characterized by a relevant shortage of human resources [5,7]. According to the Egyptian governmental mental health system assessment in 2018, there were 705 registered psychiatrists, 117 psychologists, and 224 social workers serving a population of nearly 100 million citizens [8]. In contrast, the World Health Organization recommends that the total number of psychiatrists, psychologists, and social workers in Egypt be about 5600, 24,000, and 1600, respectively [9]. Additionally, there is not only a relevant shortage of human resources, but also an unequal distribution of these resources throughout the country, with a much higher density of psychiatrists and nurses in or around Cairo compared with the rest of the country [9]. The lack of sufficient mental health staff and their unequal distribution between rural and urban areas play important roles in the under-developed mental health system of Egypt. To increase accessibility and provide higher effectiveness with the limited available resources, the mental health care system needs a new approach.

Electronic mental health (EMH) could be a strategy for improving psychiatric mental health care in Egypt. EMH is defined as mental health services and information delivered or enhanced through the internet and related technologies, and it is an expanding field with some core components as follows: psychoeducation, lifestyle mentoring, prevention, and treatment [10-12]. These technologies can help provide quality care to individuals by improving awareness, early assessment, and targeted intervention, as well as help reduce the expenditure of the health care system [13,14]. Similarly, the use of EMH through web-based psychological interventions has been shown to be effective, especially for depression and anxiety [15]. Moreover, computerized cognitive behavioral therapy and face-to-face cognitive behavioral therapy have been found to be equally beneficial to patients [16,17]. Finally, EMH allows for immediate crisis intervention, recovery, and peer support, and can be particularly useful at targeting youth [18]. Therefore, with these clear advantages, EMH would be able to help transform the Egyptian mental health care system and compensate for the low amount of human resources. However, its feasibility and implementation depend on its acceptance by specialists and other professionals in the system.

At present, individual health care apps are available in Egypt; however, there is no public implementation of EMH in health care. Available mobile apps are typically administrative in nature and help facilitate health care services, such as booking appointments with doctors and delivering medication from the pharmacy [19]. There are minimal resources that offer video conferencing with medical professionals, and those that are available have a fee-for-service model [20]. Given the increasing availability of smartphones and the increasing use of the internet, increasing EMH capacity is feasible within the Egyptian context. For instance, internet penetration has reached almost 50% of the population, with an estimated 49.23 million internet users in 2018 [21]. Egypt is a leader in technology and media for the Arab world, and EMH implementation is realistic and attainable [22]. However, support from health care providers is essential for widespread adoption of EMH tools.

Assessing the willingness and motivation of care providers to accept web-based mental health programs is an important initial step for the paradigm shift toward EMH. To answer this question, a survey was distributed and completed by Egyptian psychiatrists in order to assess their view of the current health care system, their readiness for EMH, and the perceived advantages, barriers, and priorities of EMH in Egypt. The core components of EMH assessed in the survey included screening, psychoeducation, prevention, assessment, skills training, treatment program, follow-up, peer support, communication with professionals, documentation, and informed decision-making. We aimed to better understand the opinions of psychiatrists on the current state of health care services in Egypt, their knowledge of eHealth, a topic which is not currently in the curriculum of any medical school, and their perception of how EMH could be used to improve the current system of care. Results from this survey would help conceptualize an effective, attractive, and appropriate web-based service in order to encourage a paradigm shift toward EMH within the Egyptian health care system. This survey is the first to examine the attitudes of care providers toward web-based interventions and EMH in Egypt.

## Methods

### Survey Design

The survey was developed by mental health researchers from the University of British Columbia and Tanta University, based on pre-existing questionnaires, expert opinions, and pilot testing by members of the research team. The preliminary survey was subsequently used in a workshop with 50 psychiatrists at Tanta University during a conference in March 2019 for feedback and criticism from the target population. Based on their responses, some questions were modified in order to improve the overall quality of the questionnaire and increase the legibility and coherency of the tool. The study received approval from the Tanta University Ethics Board (31674/07/17).

### Recruitment

We contacted 640 psychiatrists who were on the mailing list of the Egyptian Psychiatric Association and the Tanta University Psychiatric Department. Survey response was also encouraged during the annual psychiatric conference hosted by Tanta University in collaboration with the Egyptian Psychiatric Association. Given the number of registered psychiatrists in Egypt (less than 800 in 2018), the mailing list coverage was extensive and reached almost all registered psychiatrists within the country [8]. They were provided with an online link that directed them to the questionnaire hosted on Qualtrics Surveys [23]. Participants did not receive any reimbursement for their participation, which was entirely voluntary and anonymous. Information regarding the study purpose, research team, data collection, and confidentiality were included in the electronic consent form, which participants needed to provide before proceeding to the questions.

### Survey

The survey consisted of 36 items organized within 16 questions (Multimedia Appendix 1). Depending on participants’ previous answers, they may be asked less than 16 questions owing to skip logic. The survey was in English, as medical education in Egypt is delivered in English [24]. The internal consistency of the survey was measured by Cronbach α, which showed acceptable reliability (α=.81). The survey took between 10 and 15 minutes and consisted of both quantitative and qualitative questions organized into five broad domains as follows: (1) demographics; (2) EMH literacy and frequency of technology use; (3) efficiency of the current system of care for youth; (4) readiness to integrate EMH into the health care system; and (5) perceived priorities and barriers of EMH in Egypt. Only three questions out of 19 were open-ended, which allowed participants to elaborate on the possible drawbacks and priorities of EMH and their personal preferences for EMH in Egypt. All other questions were closed-ended and were presented as 5-point Likert scales or multiple-choice questions.

### Data Collection

The survey was available online on March 28, 2019, and kept active for 3 months. In order to avoid duplication, participants who had completed the survey were unable to submit responses again. Responses were collected electronically by Qualtrics Surveys and stored in a password-protected account. The data were stored and managed according to the data protection guidelines of the University of British Columbia. Throughout the study, the privacy of the participants was respected, and all data gathered were strictly confidential and completely anonymous.

### Data Analysis

Chi-square and one-way analysis of variance tests were performed for inferential analyses using a significance level of .05. Descriptive and inferential statistical analyses were performed using SPSS 25 (IBM Corp). The results from the web-based survey have been reported according to the Checklist for Reporting Results of Internet E-Surveys (CHERRIES) [25].

## Results

### Sample

The web-based survey was sent to 640 psychiatrists in Egypt, and of these, a total of 188 completed the survey (29.4% response rate, 90% completion rate). Given that there are less than 800 registered psychiatrists in Egypt, this sample represents nearly 25% of all registered psychiatrists in Egypt. More than half (102/188, 54.3%) of the participants were between 30 and 45 years old, while 27.7% (52/188) of the participants were over 45 years old and 18.0% (34/188) were under 30 years old. The sample was quite evenly split between females and males (54.3% [102/188] of the participants were female and 45.7% [86/188] were male).

### Technology and EMH

Of the 158 participants who responded about technology use, most said they used technology either “once daily” or “several times a day” in their everyday life (125/158, 79.3%) and at work (85/158, 53%) (Table 1). Younger participants used technology in their daily lives and at work significantly more than older participants (*P*<.001 and *P*=.02 respectively).

Of the 155 participants who responded about the efficiency of the current system and integration of EMH, only 69 (44.4%) perceived the system of care for mental health as either “extremely efficient” or “somewhat efficient” and only 44 (28.3%) reported it to be efficient for youth (Table 2). No statistical differences were found with regard to age or gender. Over half (81/155, 52.2%) of the participants said that they had current understanding and knowledge about EMH, and almost all (147/155, 94.8%) of the participants agreed that it would be beneficial for patient care. The vast majority (118/155, 76.2%) of the participants either “strongly agreed” or “somewhat agreed” with the idea of integrating EMH into the health care system (Table 2). With regard to technology use, the majority (138/155, 89.0%) of the participants agreed that the internet would be a good addition to face-to-face therapy for psychoeducation and psychosocial interventions. Additionally, 69.7% (108/155) of the participants said that they would integrate web-based mental health resources with conventional therapy if some trustworthy national internet platform for mental health and substance is present. Finally, almost all (145/155, 93.5%) of the participants said that web-based mental health resources would be useful in delivering mental health care to youth, specifically owing to their use of technology and familiarity with it.

**Table 1:** Technology use (N=158).

Characteristic			Value, n (%)		Age (years), mean (SD)
**Use of technology in everyday life**					
	Not at all	2 (1.2%)		70.0 (0.0)	
	Rarely	6 (3.7%)		44.0 (21.6)	
	Several times a week	25 (15.8%)		50.9 (9.2)	
	Once daily	35 (22.1%)		43.2 (9.3)	
	Several times a day	90 (57.2%)		37.5 (8.8)	
**Use of technology at work**					
	Not at all	9 (5.7%)		47.3 (18.5)	
	Rarely	10 (6.3%)		49 (16.9)	
	Several times a week	54 (34.2%)		42.4 (11.3)	
	Once daily	35 (22.2%)		43.9 (8.6)	
	Several times a day	50 (31.6%)		36.7 (8.1)	

**Table 2:** Efficiency of the current system and integration of electronic mental health (N=155).

Characteristic		Value, n (%)
**Efficiency of the mental health care system**		
	Extremely inefficient	17 (10.9%)
	Somewhat inefficient	41 (26.5%)
	Neither inefficient nor efficient	28 (18.2%)
	Somewhat efficient	63 (40.6%)
	Extremely efficient	6 (3.8%)
**Efficiency of the system of care for youth**		
	Extremely inefficient	35 (22.6%)
	Somewhat inefficient	50 (32.3%)
	Neither inefficient nor efficient	26 (16.8%)
	Somewhat efficient	39 (25.1%)
	Extremely efficient	5 (3.2%)
**Integration of EMH^a^ in the mental health care system**		
	Strongly disagree	1 (0.6%)
	Somewhat disagree	18 (11.6%)
	Neither agree nor disagree	18 (11.6%)
	Somewhat agree	79 (51.0%)
	Strongly agree	39 (25.2%)

^a^EMH: electronic mental health.

**Figure 1:**
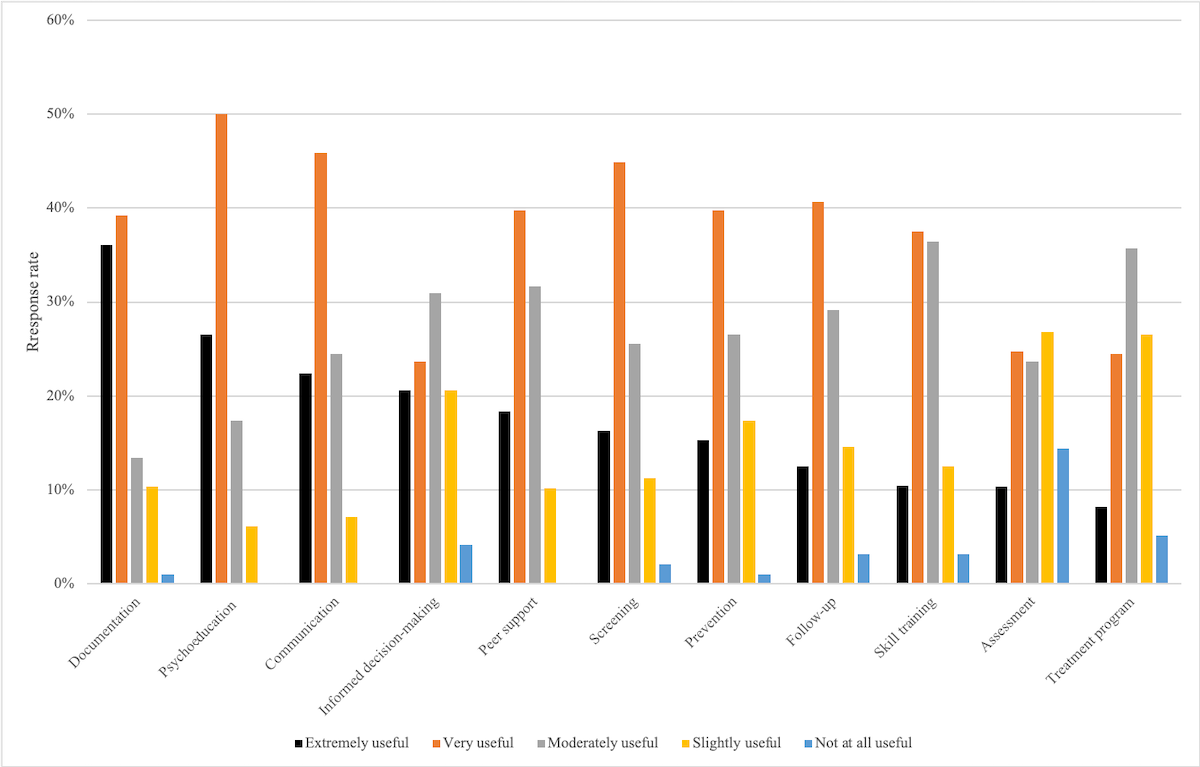
Perceived importance of certain applications of web-based mental health resources.

The perceived areas of health care that could benefit the most from EMH are shown in Figure 1. Out of the 150 participants who answered these questions, EMH was thought to be extremely useful for documentation (55/150, 36.7%), psychoeducation (40/150, 26.7%), and communication (34/150, 22.7%).

### Advantages and Drawbacks

**Figure 2:**
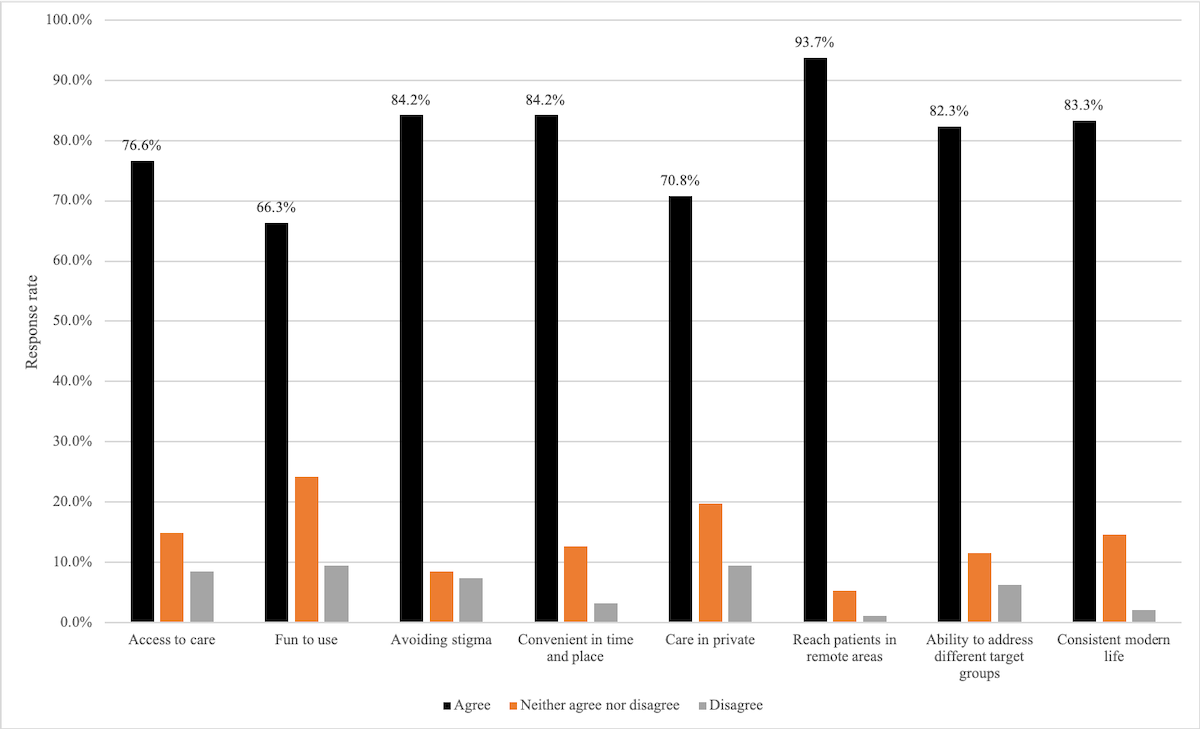
Perceived importance of certain advantages of web-based mental health resources.

The perceived advantages of web-based mental health resources are illustrated in Figure 2. The most prevalent advantage of EMH reported by those who answered these questions (N=145) was the ability to reach patients in remote areas, with 93.8% (136/145) of the participants strongly or somewhat strongly agreeing. The ability for EMH to help avoid stigma (122/145, 84.1%) and be consistent in time and place (122/145, 84.1%) were highly valued strengths as well.

The drawbacks of using web-based mental health resources were collected with open response forms and grouped together (97 participants answered this question). The possibility that personal data would be acquired by hacking the system was an important drawback, as mentioned by 27.8% (27/97) of the participants. Similarly, 20.6% (20/97) of the participants thought that the internet did not offer good information in Arabic, and 17.5% (17/97) believed that the high level of illiteracy in Egypt would hinder the implementation and use of EMH. Difficulty connecting to the internet (14/97, 14.4%) and technological problems (20/97, 20.6%) were also mentioned as drawbacks to EMH. Finally, many (16/97, 16.5%) participants believed that web-based mental health resources would negatively affect the rapport between patients and caregivers.

### Priorities

The participants were also asked in an open question to list what domain of mental health should be prioritized by web-based resources (113 participants answered this question). The majority of the participants thought that EMH should be geared toward helping mental health in youth (70/113, 61.9%) and children (32/113, 28.3%). Other domains that were reported as benefitting from EMH included substance use (23/113, 20.4%), geriatric mental health (23/113, 20.4%), suicide management (17/113, 15.0%), and personality disorders (14/113, 12.4%).

## Discussion

### Principal Findings

In our study, we explored the perspectives of Egyptian psychiatrists with regard to the state of health care services in Egypt and their interest in web-based mental health technologies. Our findings showed that the minority of participants believed that the current health care system was efficient and that almost all participants believed EMH to be a promising solution.

The findings suggest that psychiatrists in Egypt do not believe the current mental health care system in Egypt is properly equipped to meet the demand. Their perspectives are not unfounded, as several studies have also demonstrated the lack of accessible mental health resources [8]. The lack of government funding toward mental health and the high rate of physician emigration are factors that explain the deficiencies observed within the Egyptian health care system. In 2014, in Egypt, less than 1% of the total health care budget was allocated to mental health services, and about 60% of mental health services were being paid out of pocket [26]. Additionally, it is estimated that 110,000 of the 220,000 registered physicians have emigrated outside of Egypt [27]. In these situations, individuals are more likely to seek informal resources that are more accessible, such as traditional healers, religious consultants, and family members, rather than formal resources [1].

A great deal of interest for web-based solutions was expressed by the psychiatrists in our study, as almost all respondents said that these solutions would be beneficial for patient care. This was demonstrated by a trial launched in 2015 within Arabic countries called Shezlong, which allowed qualified therapists to provide web-based psychotherapy anonymously, and over 14,000 individuals sought treatment through online classrooms and video conference sessions only a year after its launch, half of which were from Egypt [28]. This staggering demand for mental health resources in Egypt is intersecting with the growth of web-based interventions in psychiatry and psychotherapy globally [29]. Medical experts have recognized that web-based interventions could lower the accessibility threshold and provide useful psychoeducation for patients [30].

Most respondents also reported they would integrate web-based solutions with their conventional therapy if a reliable internet platform is present. This is in line with previous findings that have discouraged technology as a replacement for traditional therapy, but it has been considered a new way of supporting mental health treatment [31]. Similarly, reliable eHealth solutions have been implemented in the national health systems of countries, such as Norway, Sweden, and Australia, which can provide Egypt with a blueprint [29,32]. Though resistance to change has been a major obstacle to the development of telemedicine and eHealth in Egypt, the coronavirus disease has demonstrated the utility of virtual care, which has been called into action as an emergency response to the crisis [33,34]. The Ministry of Health has designated a special hotline to provide psychological support to Egyptians isolated at home in response to the pandemic [14,35]. The adaptation to the crisis and the positive attitudes reported by the psychiatrists in our survey are promising for the implementation of web-based approaches for therapeutic interventions in Egypt.

In relation to the advantages of EMH as reported by the psychiatrists in our sample, the ability of EMH to reduce the physical and societal barriers to care was the most prevalent response. Similar advantages have been reported in the literature, including the potential for greater coverage, particularly for individuals in remote areas, and the capability to anonymously access services in order to avoid stigmatization and negative public perceptions surrounding mental illness and treatment [7,36,37]. Conversely, our findings revealed that psychiatrists believed privacy, confidentiality, and security to be the most important barriers to the implementation of EMH, similar to other studies [30,38]. Similarly, the lack of reliable content in Arabic has been shown to be a common barrier to effective web-based interventions in Middle Eastern countries and is reflected by our findings [36,39]. Cultural incompatibility in the form of differing gender-related norms, public awareness, and stigma of mental disorders, as well as language and presentation of distress are important factors to consider before using Western-developed interventions in the Middle East. It has been reported that the popularity of traditional healers in Egypt is in part a result of the comprehensive and holistic approach they offer by encompassing the patient’s spiritual beliefs, cosmology, and world view [40]. Though psychiatric services bring tremendous value to the improvement and treatment of mental illnesses, they sometimes fail to provide care within the patient’s broader cultural framework [6].

### Implications

From the psychiatrists who were surveyed in this study, our findings demonstrate that online technology seems to be an interesting and promising avenue for mental health care, not as a standalone program but as an adjunct to traditional psychotherapy. However, the implications of integrating EMH into the broader health care system will reveal challenges that will need to be addressed when necessary. In effect, at the 9th Annual Canadian E-Mental Health Conference, emphasis was placed on trust and transparency in the development of digital mental health technologies and on equity, inclusion, and access in the implementation of EMH services [41]. In developing and emerging countries, integrating new technologies, acquiring sustainable funding, and coordinating the diverse stakeholders are seen as the biggest challenges to the reorganization of the system of care [42]. However, the lack of mental health resources in Arab countries has created a surge of innovative approaches to increase access. In Lebanon, EMH has been included in the national mental health strategy. In parallel, the World Health Organization has produced a web-based intervention called “Step-by-Step” to treat symptoms of depression, which is currently being tested in Lebanon with cultural and contextual adaptations [36]. Similarly, in Saudi Arabia, a 10-year national eHealth program was launched in 2011 with the aim of developing clinical automation, telecommunication infrastructure, and a nationwide eHealth record system [43]. In Egypt, the use of online consultations in specific fields, such as dermatology and psychiatry, is starting to develop [28,44]. This is promising for the health care systems of all Arab countries, and integrating EMH seems feasible, especially given the internet penetration and mobile ownership. Egypt is among the countries with the highest proportion of mobile phone users, with around 94% of the population subscribed to a mobile phone network, and the number of Egyptian internet users reached 50 million in 2019 [21]. Health-related internet searches are also increasing (from 29.7% in 2012 to 32.4% in 2013) [45]. Despite these findings, the content of physical and mental health websites can sometimes be incomplete or inadequate [46]. A reliable online public platform for Egyptians would be beneficial for increasing mental health literacy through pertinent and up-to-date information [47].

### Limitations

The study sample (188 participants) represents over 20% of all registered psychiatrists in Egypt (around 800). Though the response rate was low for the study (28%), the findings provide valuable insights into the opinions of one in every five psychiatrists within the entire country. Similarly, although these findings are not generalizable to psychiatrists in other Arab countries or to other mental health care professionals in Egypt, the study calls for further research to look into the acceptability and use of EMH within other developing and emerging countries. Despite our findings, the participants’ responses were self-reported and could not be verified. Similarly, a web-based questionnaire by nature produces sampling bias, as we are not able to collect data from those who do not use the internet or check their emails regularly. Given the internet penetration in Egypt, this appears to be a relatively minor limitation, but one nonetheless. Within the survey itself, the validity was never tested. However, ambiguous terms, such as EMH and technology, were defined for the participants. The questionnaire was also developed through substantial pilot testing within our target population, and any misleading terminology was replaced. Finally, not many questions about the participants’ background were included, mainly because this survey was intended to be brief. If the survey had included more items, the response rate might have been even lower.

### Conclusions

Modern technology presents an opportunity to transform mobile phones into devices that can provide more cost-effective and accessible mental health care wherever and whenever it is needed. We found that Egyptian psychiatrists believed that the current mental health care system is not meeting the demand and that utilizing EMH as an adjunct to the traditional system of care would be of great interest to them. As a solution to the limited mental health resources currently available, a reliable web-based platform could take advantage of the high internet penetration and mobile ownership to increase the accessibility of psychiatric interventions throughout the country. Future research should assess the perspectives of other mental health care professionals toward web-based platforms, as well as the steps required to facilitate the development and integration of EMH within the current system of care. Finally, assessing the mental health care needs of the population, and especially those of the youth, will ensure that web-based interventions are patient-centered and effective.

## Appendix

Multimedia Appendix 1 Survey assessing the perspectives of Egyptian psychiatrists toward electronic mental health.

## Abbreviations

CHERRIES: Checklist for Reporting Results of Internet E-Surveys
EMH: electronic mental health
