# Supplementary material for: Electronic Mental Health as an Option for Egyptian Psychiatry: Cross-Sectional Study
Source: JMIR Ment Health. 2020 Aug 13;7(8):e19591. doi: 10.2196/19591 (PMC7453323; doi:10.2196/19591)
Supplement: Multimedia Appendix 1 [file mental_v7i8e19591_app1.pdf]

SUBJECT INFORMATION AND CONSENT FORM

Principal Investigator:
Mostafa Mamdouh, MD, Ph.D. candidate
Department of Psychiatry, Tanta University.

Addictions & Concurrent Disorders (ACD) Group
Department of Psychiatry, Faculty of Medicine
University of British Columbia, Canada.

Purpose: We would like to invite you to take part in our research survey. We are seeking to understand caregivers' opinions and perception of current services and how e-health can be beneficial to mental health in Egypt in order to help improve the current system of care for mental health in Egypt.

Your participation is voluntary: Your participation is entirely voluntary – it is up to you to decide whether or not to take part in this study. Before you decide, it is important that you understand what this research involves. This consent form will tell you about the study and your involvement. If you decide to participate, you will be asked to sign a consent form by clicking I agree. You are free to withdraw at any time and may choose not to participate at all, without giving any reasons for your decision.

Who can take part in this study? You can participate in this study if you are a registered psychiatrist.

Study procedures: If you decide to take part in this study, you will complete a series of questions delivered to you through this online survey. At any time, if there is a question you are not comfortable with, you do not have to answer it and can skip to the next question. This survey consists of about 20 questions will take approximately 5 to 10 minutes to complete in a single session. *This study has received ethical approval by the Tanta university research ethics board.*

Compensation: Your participation is voluntary and there will be no reimbursement provided for this study.

Potential benefits: You may not directly benefit from your participation in this study. However, we hope to use this information to improve our system of care for mental health.

Confidentiality:
Throughout the entire study, we will respect your confidentiality. All information gathered from this survey will remain completely anonymous.

Contact for information about the study: If you have any questions or want more information about this study, please contact Mostafa Mamdouh at this email: mostafamamdouh17@gmail.com

Subject consent to participate:
By clicking "I agree", you consent to participate in this project. This statement certifies the following: I have read and understood the subject information and consent form. I understand that all of the information collected will be kept confidential and that the result will only be used for scientific objectives. I understand that my participation in this study is voluntary and that I am completely free to

refuse to participate or to withdraw from this study at any time. I agree and consent to participate in this study.

- ○ I agree and consent to participate in this study.
- ○ I do not agree and consent to participate in this study.

Skip To: End of Survey If "Survey of E-mental health feasibility for mental health specialists" SUBJECT INFORMATION AND CON... = I do not agree and consent to participate in this study.

Our system of care needs to address ongoing change. In order to be able to increase quality and efficacy, mental health specialists need to be proactive and be actively involved in system reform. We invite you to share your thoughts in this survey.

1. **What's your age?**

[Dropdown list]

2. **What's your gender?**

- ◯ Male
- ◯ Female

3. **On a scale from 1 to 5, how often do you use technology in your daily life and for work?**
Technology defined as the internet, computer, laptop, etc.

	1<br>not at all	2<br>Less frequently	3<br>Several times a week	4<br>Once daily	5<br>Several times a day
For daily life?	◯	◯	◯	◯	◯
For your work?	◯	◯	◯	◯	◯

4. **In your opinion, how efficient is the current system of care for general mental health and substance use, as well as for youth?** Efficiency defined as general accessibility, non-stigmatizing, ability to address different target groups and reach patients in remote areas, etc.

	Extremely inefficient (1)	Somewhat inefficient (2)	Neither efficient nor in efficient (3)	Somewhat efficient (4)	Extremely efficient (5)
Mental health and substance use	○	○	○	○	○
Youth mental health and substance use	○	○	○	○	○

5. **Do you know about E-Mental Health?**

○ Yes

○ No

E-Mental Health (EMH) is defined as mental health services and information delivered or enhanced through the internet and related technologies. Some of these services, such as video conferencing or online counseling, involve direct one-on-one contact with a mental health professional. Other e-mental health services, such as web applications or information websites, involve less or no contact with mental health professionals.

6. **Do you think EMH may be beneficial in your field for patient care?**

○ Yes

○ No

7. **On a scale from 1 to 5, how useful do you think EMH can be for the following:**

	Not at all useful (1)	Slightly useful (2)	Moderately useful (3)	Very useful (4)	Extremely useful (5)
Screening	○	○	○	○	○
Psycho education	○	○	○	○	○
Prevention	○	○	○	○	○
Assessment	○	○	○	○	○
Skill training	○	○	○	○	○
Treatment program	○	○	○	○	○
Follow-up	○	○	○	○	○
Peer support	○	○	○	○	○
Communication with professionals	○	○	○	○	○
Documentation	○	○	○	○	○
Informed decision making	○	○	○	○	○

8. **On a scale from 1 to 5, to what extend do you agree with the following: EMH can be advantageous because of**

	Strongly disagree (1)	Somewhat disagree (2)	Neither agree nor disagree (3)	Somewhat agree (4)	Strongly agree (5)
access to care	○	○	○	○	○
fun to use	○	○	○	○	○
avoid stigma	○	○	○	○	○
convenient in time and place	○	○	○	○	○
care in private (privacy)	○	○	○	○	○
reach patients in remote areas	○	○	○	○	○
ability to address different target groups	○	○	○	○	○
consistent with our modern lives	○	○	○	○	○

9. **On a scale from 1 to 5, to what extend do you agree with the following: web-based interventions should be:**

	Strongly disagree (1)	Somewhat disagree (2)	Neither agree nor disagree (3)	Somewhat agree (4)	Strongly agree (5)
integrated as a part of our system of care.	○	○	○	○	○

10. **If there was a reliable national internet platform for mental health and substance use, would you prefer it to conventional therapy for your patients?**

◯ Yes

◯ No

11. **In addition to face-to-face therapy, is it practical to use an accompanying psychoeducational or psychosocial or additional intervention via the internet?**

◯ Yes

◯ No

12. **Considering that technology is frequently used by youth in Egypt, do you think is it a good idea to use technology in delivering mental health care to them?**

◯ Yes

◯ No

13. **Do you think there are drawbacks to using EMH in Egypt?**

◯ Yes

◯ No

Skip To: 16 If Do you think there are drawbacks to using EMH in Egypt? = Yes

Skip To: 17 If Do you think there are drawbacks to using EMH in Egypt? = No

Display This Question:

If Do you think there are drawbacks to using EMH in Egypt? = Yes

14. **What are possible drawbacks?**

____

____

____

____

____

15. **What are 3 ways in which you wish to see web-based resources used in the next 3 years?** Online cognitive behavioral therapy, prevention, assessment, etc.

____

____

____

16- **What should be the 3 priorities in the development of EMH?** Specific population, specific interventions, specific illness, etc.

____

____

____

____

____
